# Royalty and St. Bartholomew's

**Published:** 1921-02-19

**Authors:** 


### Royalty and St. Bartholomew's.

It may be premised that although on the authority of *Liber fundacionis ecclesie sancti Bartholomei* St. Bartholomew's Hospital and Priory were erected in 1123 on land granted to Rahere, the founder, by King Henry I., there is no contemporary evidence of the fact. In the Itinerary of Henry I., recently published in the *English Historical Review*, no mention is made either of Rahere or the priory. Copies of the royal charter of 1133 mention the hospital, but that charter concerns itself chiefly with the priory, to which, amongst other privileges, it grants the right of holding a fair. By it Henry undertook to maintain and defend this church even as his crown.

But at the Dissolution the connection of the King's name with the hospital was at first not very promising, and by that Act it passed into the king's hand in 1537. A petition of the Corporation of the City of London was presented to Henry VIII. in 1538 asking that St. Bartholomew's, St. Thomas's, and Bethlehem should be given to them, but it was not until 1546 that the petition, so far as St. Bartholomew's Hospital was concerned, had the desired effect. On June 23, 1544, by letters patent, the hospital was restored and refounded under the control of a master, a priest, and four chaplain priests, and at the end of 1546 the king granted the hospital to the mayor and citizens of London, endowed it with an estate of five hundred marks, and constituted it a parish to be known as Little St. Bartholomew in West Smithfield.

### The Nineteenth Century.

There is little specially to connect St. Bartholomew's Hospital with Royalty between this date and the nineteenth century, and from that time relations became much more intimate. On January 1, 1845, H.R.H. the Prince Consort was invested with the green staff of office as a governor; the customary ceremony was performed at Windsor Castle by the president, three aldermen, and the clerk of the hospital. This event was commemorated by the presentation to the hospital by the president and treasurer of three pieces of handsome silver plate—on the first was depicted Prince Albert receiving his staff as governor, on the second a representation of the Good Samaritan, and on the third a design illustrative of the Plague of London.

In 1869 Queen Victoria and Princess Louise, attended by Sir William Jenner, visited several of the wards of the hospital and conversed with the patients. At the same time, as Prince of Wales, King Edward VII. took a prominent part in the hospital's affairs. His Royal Highness was elected President on March 20, 1867, and he lost no time in visiting his charge, for on April 3 of that year, accompanied by the King and Queen of Denmark, he made a thorough tour of the institution. The following week, on April 10, he was installed in the presidential chair.

In 1877 a reconstruction of the medical school was decided upon, and on May 3, 1879, the Prince, accompanied by the Princess, performed the opening ceremony in the Museum and later partook of luncheon in the Great Hall. Two years later, on May 27, 1881, he and the Princess attended a conversazione to celebrate the completion of the school, and on July 3, 1885, the Prince and Princess opened the Convalescent Home at Swanley in Kent, which had been presented to the hospital by Mr. C. T. Kettlewell. Ten years later the Prince, accompanied by the Crown Prince of Denmark, was present at the consecration of the Rahere Lodge of Freemasons in the Great Hall of the hospital; and the last time His Royal Highness presided over a Court of Governors was in July, 1900, when the momentous question of acquiring additional site was considered. In the following January Queen Victoria died, and on his accession to the throne the Prince resigned the office of President which he had held for thirty-four years.

### Our Present King.

On December 4, 1901, H.R.H. the Duke of Cornwall and York was formally installed as President of the hospital in succession to his august father, and on February 15, 1902, accompanied by his consort, inspected the hospital. In 1904 Edward VII. and Queen Alexandra came in state to lay the foundation-stone of the new out-patient department. The opportunity was taken to admit the Queen as a Governor; Her Majesty was thus the first lady to hold a Governorship of the hospital. In place of the ordinary green staff bearing the arms of the hospital which it is customary to present to every Governor, Her Majesty was pleased to accept a miniature staff of silver, enamelled in green. Later, on July 23, 1907, the Prince and Princess of Wales formally opened the out-patient buildings, and the Princess also received her charge and staff of office as a Governor.

### H.R.H. The Prince of Wales.

In 1910, when King Edward VII. died and was succeeded by the Prince of Wales, the latter relinquished the Presidency of the hospital and became Patron. From that time until the present Prince of Wales became President in February, 1920, the office remained vacant.

On November 7, 1914, the King and Queen visited the hospital, and were conducted round the wards, which had been placed at the disposal of the War Office. They spent two hours in conversing with the wounded soldiers on this occasion, and later on several occasions Royalty visited victims of the air-raids who were being treated at St. Bartholomew's.

It was on February 19, 1920, that H.R.H. the Prince of Wales was made a Governor, and installed as President of the hospital in the Great Hall. Afterwards he made a tour of inspection of the hospital.

